# Cross-disease communication between cancer and heart failure provides a rational approach to prevention and treatment of both diseases

**DOI:** 10.3389/fonc.2022.1006322

**Published:** 2022-10-31

**Authors:** Shingo Takada, Shintaro Kinugawa, Haruka Handa, Takashi Yokota, Hisataka Sabe

**Affiliations:** ^1^ Department of Lifelong Sport, School of Sports Education, Hokusho University, Ebetsu, Japan; ^2^ Department of Molecular Biology, Hokkaido University Graduate School of Medicine, Sapporo, Japan; ^3^ Department of Cardiovascular Medicine, Faculty of Medical Sciences, Kyushu University, Fukuoka, Japan; ^4^ Division of Cardiovascular Medicine, Research Institute of Angiocardiology, Faculty of Medical Sciences, Kyushu University, Fukuoka, Japan; ^5^ Institute of Health Science Innovation for Medical Care, Hokkaido University Hospital, Sapporo, Japan; ^6^ Institute for Genetic Medicine, Hokkaido University, Sapporo, Japan

**Keywords:** immune-checkpoint inhibition, mitochondrial oxidative phosphorylation, disease prevention, myokine, PBMC, exercise, diet, reactive oxygen species

## Abstract

Accumulating clinical data have demonstrated a clear positive association between cancer and cardiac disorders, particularly chronic heart failure (CHF). These two diseases can be mutual drivers of each other, and hence frequently co-occur in patients. The immune system is the core mechanism that eliminates transformed cells from our bodies. However, immune cells often play distinct or even conflicting roles in cancer and CHF. Moreover, CHF alters the properties of immune cells, particularly those of regulatory T cells. Our previous study showed that the oxidative phosphorylation capacity of peripheral blood mononuclear cells is impaired in CHF, leading to the increased production of reactive oxygen species. Therefore, the co-occurrence of cancer and CHF becomes a serious problem, affecting the treatment of both diseases, and consequently negatively affecting patient survival rates. To date, few methods have been identified that effectively treat both diseases at the same time. Mitochondria activity may change in immune cells during their activation and exhaustion, and in CHF. Mitochondria activity is also largely affected in myocardia in CHF. We here focus on the mitochondrial abnormalities of immune cells in cancer and CHF, and discuss possible ways to treat cancer and CHF at the same time by targeting mitochondrial abnormalities. Many cancer cells are inevitably produced daily in our bodies, mostly owing to enzymatic nucleotide errors of DNA replication and repair. Therefore, the possibility of ways to prevent cancer by preventing the onset of heart failure will also be discussed.

## Introduction

**Figure 1:**
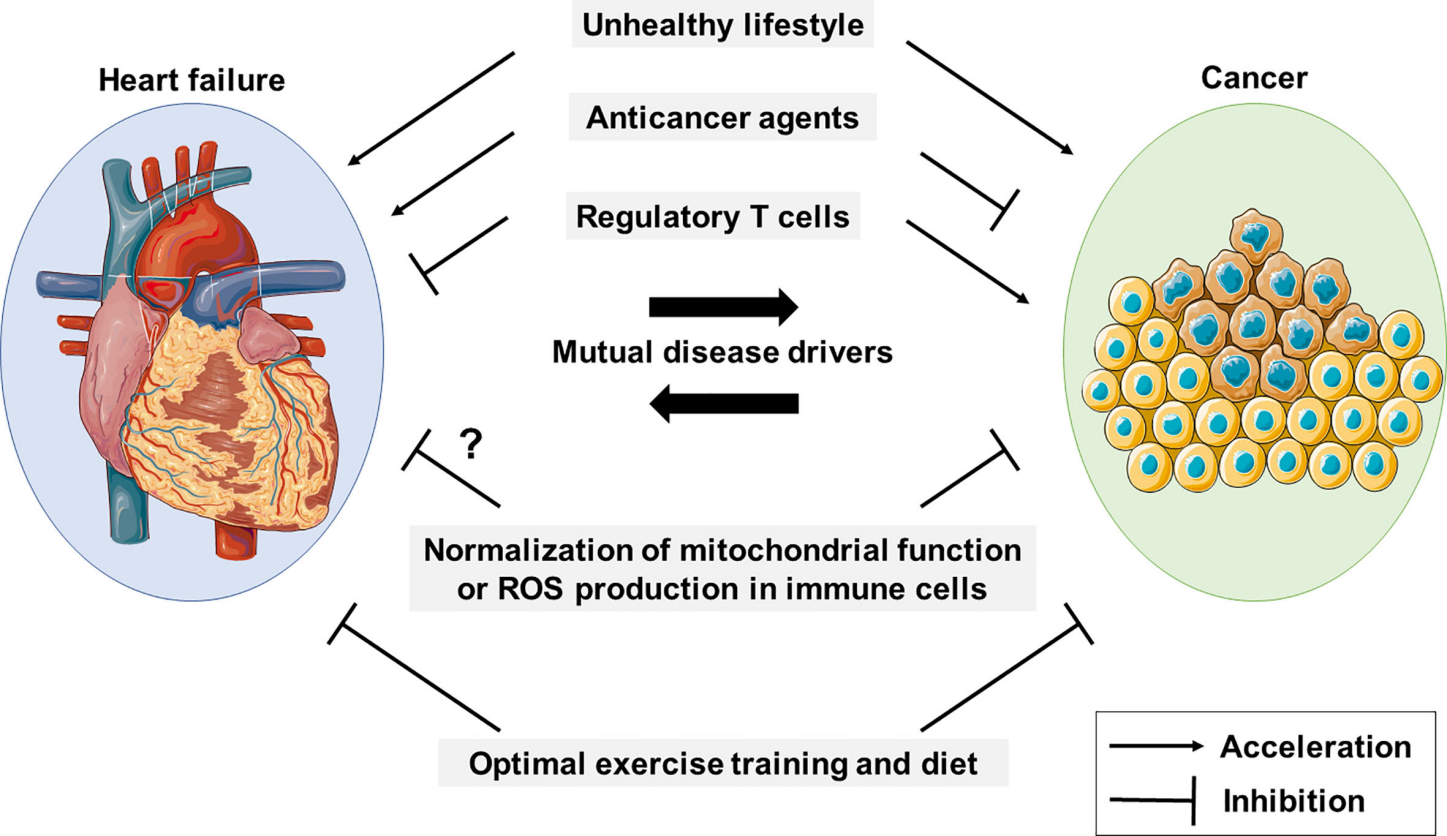
Cancer and HF mutually promote each other. An unhealthy lifestyle contributes to the development of cancer and CHF, and they are mutual disease drivers. Anticancer drugs and regulatory T cells appear to have conflicting roles in cancer and HF. Mitochondrial function and reactive oxygen species (ROS) production in immune cells are potential therapeutic targets in both diseases.

Cancer and cardiac disorders, including chronic heart failure (CHF), represent two major causes of morbidity and mortality in developed countries (1, 2). Epidemiological studies have shown that the risk of developing cancer in patients with CHF is approx. four times greater than in those without CHF (3–6). Conversely, cancer patients can be at increased risk of cardiac disease due to deterioration of their lifestyle behaviors (*e.g.*, inactivity and an unbalanced diet) (7), and also due to treatment toxicity, as many anticancer drugs are known to cause cardiotoxic side effects (8–11). Therefore, cancer and cardiovascular diseases can be mutual disease drivers, and hence co-occur frequently in patients (**Figure 1**). Moreover, immune cells, particularly regulatory T (Treg) cells, play distinct or even conflicting roles in cancer and CHF (12, 13). Hence, the co-occurrence of cancer and cardiovascular disease is a serious problem, affecting the treatment of both diseases, and consequently negatively affecting survival rates (14, 15). To date, however, treatments exist only for each disease. Mitochondria are central to ATP production by oxidative phosphorylation (OXPHOS) and to metabolism. To address above problems, we here focus on the mitochondrial abnormalities of immune cells during CHF and cancer, and discuss possible methods to treat cancer and CHF at the same time by targeting these mitochondrial abnormalities; and, moreover, discuss possible ways to prevent cancer by preventing the onset of CHF.

## Immune system mediates the crosstalk between cancer and CHF

T-cell dysfunction, particularly of tumor-infiltrating lymphocytes (TILs), is highly detrimental to antitumor immunity and immunotherapy (16). Recently, Koelwyn et al. reported that the adjusted relative risk of death from breast cancer is increased by approx. 60% in the presence of a cardiovascular event (17). They also demonstrated by using mouse models that myocardial infarction (MI), which leads to HF, accelerates breast cancer development (17). Molecularly, it was shown that MI epigenetically reprogrammed Ly6C^hi^ monocytes, which are macrophage precursors in the bone marrow reservoir, to an immunosuppressive state, and increased their circulation and infiltration into tumors, whereas their depletion abrogated tumor growth (17). Moreover, tumors of MI mice had fewer T lymphocytes than control mice, in which T_reg_ cells are predominant. These changes that occur in MI mice may be beneficial to the heart, but they all promote tumor growth and survival (17). Therefore, certain populations of immune cells clearly play a central role in cross-disease communication between cancer and CHF.

## CHF affects mitochondrial OXPHOS of immune cells

Mitochondrial OXPHOS plays a central role in lymphocyte activity (18). Mitochondria are also fundamental to the development and fate determination of peripheral lymphocytes (19, 20). Suppressed glycolysis and OXPHOS were shown to be early drivers of CD8^+^ T-cell exhaustion (21). Moreover, TILs are constantly exposed to tumor antigens, and may also experience metabolic stress, which is thought to occur frequently in the tumor microenvironment. A recent report demonstrated that continuous antigen stimulation together with hypoxia impairs the mitochondrial functions of T cells, and hence promotes terminal T-cell exhaustion (22). Molecularly, it was shown that continuous antigen stimulation upregulates B lymphocyte–induced maturation protein 1, and represses peroxisome proliferator-activated receptor gamma coactivator-1 (PGC-1), resulting in the suppression of mitochondrial biogenesis and T-cell functions (23).

Our group has found that mitochondrial respiratory capacity of peripheral blood mononuclear cells (PBMCs), which are predominantly lymphocytes, declines with the progression of CHF, with class III (i.e., moderate to severe CHF) patients by New York Heart Association (NYHA) criteria having 10-24% lower mitochondrial respiratory capacity than NYHA class I/II (i.e., mild CHF) patients, in which mitochondrial ROS production of PBMCs was increased by 13-24% in patients with NYHA class III compared to those with NYHA class I/II (24). Such changes were observed even in the early stages of HF, and were closely associated with the severity of CHF. We moreover found that the capacity of complex II, but not complex I, of the mitochondrial OXPHOS of PBMCs was specifically decreased in CHF (24). It has been reported in monkeys that there is a close association among the mitochondrial OXPHOS activities of circulating monocytes, cardiac cells, and skeletal muscle cells (25).Therefore, ROS levels in PBMCs can be a marker indicating the onset and the severity of HF. As PBMCs mostly consist of unprimed lymphocytes, it awaits to be clarified whether activated lymphocytes are also affected in CHF patients.

## Activating mitochondria of immune cells improves tumor immune therapies

Mitochondrial function of CD8^+^ T cells in lung cancer patients can be a marker for determining the efficacy of anti-PD-1 immune checkpoint inhibition therapy (26). Scharping et al. have shown that restoration of mitochondrial activity and T-cell function by reversing the loss of PGC-1α in tumor-specific T cells resulted in increased antitumor immune responses (23). Yu et al. demonstrated that administering nicotinamide riboside (NR), a precursor of nicotinamide adenine dinucleotide, may be able to restore mitochondrial activity, prevent T-cell exhaustion, and sustain the antitumor responses of T cells in tumor-bearing mice (27). NR supplementation was moreover found to facilitate antitumor immune activity, when used in conjunction with the anti-PD-1 antibody (27). Vardhana et al. demonstrated that N-acetylcysteine (NAC), which is known to increase glutathione synthesis and neutralize ROS, reverses the metabolic defects of exhausted T cells, and promotes their antitumor immune activity, to act synergistically with anti-PD-L1 immunotherapy in lymphoma and melanoma (28). Therefore, activating immune cell mitochondria may improve the efficacy of immune checkpoint inhibition-based tumor immunotherapy. However, it should be noted that the administration of molecules such as NR or NAC may also activate cancer cells to more malignant states, and it is hence unclear whether they will be effective in the treatment of patients. It is also well documented that the reinvigoration of T cells, once they are deeply exhausted, might be very difficult (29). Another way to improve the efficacy of cancer immunotherapy would be to enhance the new production of T cells, and diversify the T-cell receptor repertoire, as has been demonstrated with radiation (30), but this might also be difficult in patients with CHF because of their poor health condition.

## Future perspectives

When cancer and CHF coexist, the treatment of either disease alone is inadequate. Normalization of mitochondrial activity and the function of immune cells, which are frequently impaired in CHF, is a rational strategy to improve cancer therapeutics. For example, identification of a molecular basis for the downregulation of mitochondrial respiratory capacity in the PBMCs of CHF patients, which we have shown previously (24), and if such a mechanism occurs specifically in PBMCs but not in tumor cells, improving mitochondrial respiratory capacity in PBMCs may be promising for the treatment of cancer in patients who also have CHF. Such a strategy targeting immune cells’ mitochondria may also enhance tumor growth suppression in cancer treatment by immune checkpoint inhibitors, although cardiac assessment with a careful follow-up is necessary because immune checkpoint inhibitors are known to have a cardiotoxicity with low incidence rate (<1%) with single use of them (31). Furthermore, activation of immune cells is beneficial for chemotherapy (32), and thus, mitochondria-targeted treatment strategy may help chemotherapy improve outcomes of cancer patients with or without CHF, although robust clinical evidence is still lacking.

Lifestyle habits, such as a proper diet and daily exercise are important preventive measures of cancer and CHF. Regarding the molecular bases, skeletal muscles secrete various myokines, which have positive effects on mitochondria in different organs and tissues, and may also promote immunity (33–35). Proper exercise by patients can also suppress tumor growth and promote anti-tumor immunity, and may improve the therapeutic effects of immune-checkpoint inhibitors, whereas the types of myokines and immune cells therein involved have been shown to differ depending on the types of cancer (36–38). On the other hand, muscle dysfunction occurs not only in CHF (35, 39), but is also a widespread phenomenon of cancer patients regardless of cancer type or stage (40). Therefore, the identification of the singular point (41) before which exercise can be effective in the treatment of cancer and CHF, along with identification of effective exercise regimens and the related drugs, will be the major challenge of medicine in the future.

## Author contributions

ST, SK, HH, TY, and HS wrote the manuscript. All authors contributed to the article and approved the submitted version.

## Funding

This work was supported in part by Grants-in-Aid for Scientific Research (grant no. JP17H04758 to ST, and 21H03360 to SK, and 21H02675 to HS), and Grants-in-Aid for Challenging Exploratory Research (grant no. 19K22791 to ST) from the Japan Society for the Promotion of Science, grants from the Akiyama Life Science Foundation (to ST), the Suhara Memorial Foundation (to ST), and the Japan Foundation for Applied Enzymology (to ST).

## Acknowledgments

The authors thank H.A. Popiel for her critical reading of the manuscript.

## Conflict of interest

The authors declare that the research was conducted in the absence of any commercial or financial relationships that could be construed as a potential conflict of interest.

## Publisher’s note

All claims expressed in this article are solely those of the authors and do not necessarily represent those of their affiliated organizations, or those of the publisher, the editors and the reviewers. Any product that may be evaluated in this article, or claim that may be made by its manufacturer, is not guaranteed or endorsed by the publisher.
